# Factors influencing vocational nursing students’ career intentions in geriatric nursing: a cross-sectional study

**DOI:** 10.1186/s12912-026-04346-z

**Published:** 2026-01-28

**Authors:** Lizhen Liu, Xianmei Zeng, Ping Tang, Kai Qi, Ping Wang

**Affiliations:** 1 Department of Nursing, Hunan Traditional Chinese Medical College, No. 136 Lusong Road, Lusong District, Zhuzhou, 412012 China; 2 School of Nursing, Yongzhou Vocational Technical College, Yongzhou, 425100 China; 3 Xiangya Nursing School, Central South University, Changsha, 410013 China

**Keywords:** Geriatrics, Older adults, Willingness to care, Vocational education, Nursing education, Nursing students, Attitudes

## Abstract

**Background:**

The accelerating global aging population creates an urgent demand for skilled geriatric nurses, yet geriatric nursing remains an unpopular career choice among vocational nursing students in China. Vocational institutions are strategically positioned to supply the core geriatric nursing workforce. This study aimed to evaluate vocational nursing students’ career intentions in geriatric nursing in Central-South China and identify the key influencing factors.

**Methods:**

This cross-sectional study was conducted from March to June 2024, with five vocational institutions in Central-South China randomly selected using cluster sampling. Data were collected through a demographic questionnaire, the Facts on Aging Quiz 1 (FAQ1), and the Chinese version of Kogan’s Attitudes towards Old People (KAOP) Scale. Logistic regression analysis was employed to identify factors influencing students’ career intentions in geriatric nursing.

**Results:**

Of the 3622 surveyed students, 1763 (48.67%) expressed willingness to pursue geriatric nursing post-graduation. Influencing factors included sociodemographic characteristics (lower year of study, nursing as a first career choice), experiential and educational factors (positive intergenerational contact, prior practical experience, and perceived need for specialized geriatric courses), and stronger gerontological knowledge and positive attitudes toward older adults.

**Conclusions:**

Vocational nursing students’ career intentions for geriatric nursing are at a low-to-moderate level. They are shaped by sociodemographic, experiential, and attitudinal-knowledge factors. Low knowledge levels necessitate targeted interventions. Integrating gerontological curricula, expanding clinical placements, and fostering intergenerational contact will nurture motivation and enhance preparedness in this critical healthcare sector.

**Supplementary Information:**

The online version contains supplementary material available at 10.1186/s12912-026-04346-z.

## Introduction

The global population aged 65 and older is anticipated to grow to 1.6 billion by 2050, effectively doubling from current figures, as the world experiences rapid aging [1]. China faces a particularly acute demographic shift, as its older adult population is projected to soar from 172 million (12%) in 2020 to 366 million (26%) by 2050 [2]. However, this rapid aging has outpaced the development of geriatric nursing services, creating significant workforce and service delivery gaps, particularly in China [3]. This underscores an urgent need to expand the healthcare workforce to meet the complex demands of aging populations.

Nurses are the primary providers of geriatric care services. However, many countries, including China, face a critical shortage of qualified professionals, especially in nursing homes and community settings [4, 5]. Heavy workloads, low remuneration, and limited social recognition hinder recruitment and retention [6–9]. Moreover, many geriatric nursing workers are older, with lower educational levels and limited professional qualifications, which compromises care quality and safety [10]. Therefore, an increased provision of qualified nurses is imperative to fulfill the diverse requirements of the elderly population and improve care standards.

In China, vocational college nursing students account for over 60% of nursing students and are vital for addressing geriatric nursing workforce shortages [11]. They constitute a significant proportion of nursing enrollment in China and other countries with structured vocational education systems [12, 13]. Targeting high school graduates, vocational programs emphasize practical training for clinical and geriatric care roles, in contrast to undergraduate programs that focus more on theoretical education [14, 15]. This hands-on approach equips students to address the multifaceted demands of an aging populace, thereby positioning them as the backbone of China’s future geriatric care workforce. Despite their potential, vocational nursing students demonstrate limited engagement in geriatric nursing, posing a significant challenge to workforce development.

Globally, geriatric nursing is often an unpopular career choice among nursing students, with studies indicating that less than 10% of baccalaureate nursing students in countries like the United States and Australia prioritize geriatric nursing [16]. Negative perceptions, such as viewing geriatric nursing as unchallenging or emotionally taxing, exacerbated by limited clinical exposure and societal ageism, underlie vocational nursing students’ reluctance [16–18]. Similarly, in China, undergraduate students exhibit low interest in long-term care, influenced by factors including prior experiences and professional expectations [19]. These findings highlight the complex barriers deterring students from pursuing careers in geriatric care, suggesting the need for targeted educational reforms, enhanced clinical exposure, and attitudinal change interventions.

Despite extensive research on nursing career preferences among undergraduate students and practicing nurses, evidence on vocational nursing students’ career intentions in geriatric nursing remains limited, even though they constitute the core workforce for elder care in rapidly aging regions such as Central-South China. Globally, vocational nursing students also play a central role in aged care systems, including in countries like the United States and Australia, yet studies exploring their career intentions are scarce. The unique educational context of vocational programs in China, often marked by limited geriatric-specific curricula and clinical placements, remains underexplored in relation to students’ career intentions. By examining this understudied population, this study seeks to bridge a significant evidence gap and offer valuable insights for developing targeted educational interventions and workforce strategies, ultimately enhancing geriatric care capacity in China and other aging societies.

To gain a deeper understanding of the factors influencing vocational nursing students’ career intentions in geriatric care, this study utilizes Ajzen’s [20] Theory of Planned Behavior (TPB). This framework suggests that a person’s intention to perform a specific behavior is primarily influenced by their personal evaluations of the behavior, their perceptions of social pressures regarding the behavior, and their perceived ability to perform it. These constructs provide a comprehensive framework for analyzing students’ willingness to pursue geriatric nursing based on their experiences, knowledge, and attitudes.

## Methods

### Study design, settings, and participants

This study employed a cross-sectional descriptive design to examine the career intentions of vocational nursing students in geriatric nursing. It was conducted in five public vocational nursing schools in Central-South China, encompassing Henan, Hubei, Hunan, Guangdong, and Guangxi provinces. A cluster random sampling method was applied. From 48 public vocational nursing schools listed in the Ministry of Education’s registry (8–12 per province), one school per province was randomly selected using SPSS (Version 26) to ensure unbiased regional coverage.

Each school was assigned a unique numerical code, and the school corresponding to the first randomly generated number was selected. Eligible participants were full-time vocational nursing students in their first, second, or third year, who provided voluntary informed consent. Conversely, exclusion criteria included students who had withdrawn from the program, were currently suspended, were diagnosed with mental illness precluding participation, or were unable to complete the questionnaire due to language or cognitive barriers. All eligible students were invited to participate.

### Sample size

The sample size for this cross-sectional study was justified to ensure adequate statistical power and stability for the multivariable binary logistic regression analysis. The determination followed the Events Per Variable (EPV) criterion, a recommended standard for regression models [21]. Our final model included 15 predictor variables. Based on a conservative EPV ratio of 10:1, the required minimum number of events (students’ willingness to pursue geriatric nursing) was 150. By referencing an estimated event prevalence of 33.8% from prior literature [19], the theoretical minimum sample size required was calculated as 444 participants. To ensure robust representativeness and statistical power, we employed a cluster random sampling method across five provinces. This strategy yielded a final sample of 3622 students, which substantially exceeds the minimum theoretical requirement of 444, ensuring adequate model stability and power to detect small-to-medium effect sizes.

### Data collection

Data were gathered from March to June 2024 using a self-administered questionnaire distributed through Questionnaire Star (https://www.wjx.cn) and paper-based formats. Following approval from academic department heads, research assistants coordinated with course instructors to access student schedules. Questionnaires were distributed and collected during class breaks to minimize disruption. Completing the questionnaire took approximately 15–20 min. Paper-based responses were double-entered to ensure accuracy, and online responses were exported directly from Questionnaire Star.

### Instruments

#### Demographic data

Based on previous research in geriatric nursing education [19, 22], a self-designed demographic questionnaire was used to collect information on various factors, including sociodemographic characteristics, experiential factors, and educational factors. It also assessed the primary outcome variable: intention to pursue a career in geriatric nursing. Participants’ sociodemographic characteristics encompassed age, gender, year of study, residential area (urban or rural), only child at home, nursing as a first career choice when applying for college and the province where their school was located (Hubei, Hunan, Guangdong, Guangxi, Henan). Experiential factors included relationship with the elderly, living with the elderly for at least six months, primarily receiving care from grandparents during childhood, having volunteer experience with the elderly, and having prior work experience in a nursing home. Educational factors covered the perceived need for specialized geriatric nursing courses and actual receipt of geriatric nursing education. The primary outcome, intent to pursue geriatric nursing, was assessed with a single-item question: “Would you consider working with older adults upon graduation?” with response options of “Yes” or “No”. These variables were pilot-tested to ensure clarity and relevance (see Additional File 1 for the full English version of the questionnaire).

#### Aging knowledge

The Facts on Aging Quiz 1 (FAQ1), originally developed by Palmore [23] was utilized to evaluate participants’ understanding of aging. This study employed the Chinese version of the FAQ1 validated by Wang et al. [24]. This 25-item scale examines individuals’ knowledge of aging across physical, mental, and social dimensions. Participants responded to each statement as “true”, “false”, or “unsure”. For each item, 1 point was awarded for a correct identification (either “true” for factual statements or “false” for misconceptions), while incorrect or “unsure” responses received 0 points. This produced a total score range of 0 to 25, with higher scores indicating greater knowledge of aging. The Chinese adaptation of the scale showed adequate internal consistency (Cronbach’s α = 0.68) and a content validity index (CVI) of 0.82 [24]. In this study, the Cronbach’s α coefficient of the scale was 0.701.

#### Attitudes toward older adults

Students’ attitudes toward older adults were assessed using Kogan’s Attitudes towards Older People (KAOP) Scale. Originally developed by Kogan [25], the Chinese version of the KAOP validated by Yen et al. [26]. was utilized in this study. This 34-item instrument features an equal distribution of 17 positively and 17 negatively worded statements. Responses were recorded on a 7-point Likert scale ranging from 1(“strongly disagree”) and 7 (“strongly agree”). Negative statements were reverse-scored. Total scale scores range from 34 to 238, where higher scores indicate more favorable attitudes toward older adults. A median score of 136 has been reported [19]. The Chinese version of the KAOP Scale demonstrated strong internal consistency, as evidenced by a Cronbach’s alpha of 0.82 [26]. The Cronbach’s α coefficient of the scale in this study was found to be 0.812.

### Theoretical framework

This study applied TPB to examine factors influencing nursing students’ intentions to practice in geriatric settings. TPB posits that behavior is driven by three constructs: attitudes toward the behavior, subjective norms, and perceived behavioral control. In this study, attitudes were measured using KATOP Scale, which assesses students’ views on working with older adults. Subjective norms were operationalized as interpersonal and family-based social influences, including experiences such as receiving care from grandparents or maintaining relationships with elders, assessed via a self-reported questionnaire. Perceived behavioral control was evaluated through indicators such as geriatric care education, volunteering, and nursing home experience, which shape students’ confidence in performing geriatric nursing tasks. This framework guided the selection and interpretation of variables in the study.

### Data analysis

Data were imported into SPSS for analysis. Descriptive statistics were used to examine sociodemographic characteristics (e.g., age, gender, residential area, only child status, Province of school, nursing as a first career choice), caregiving experience, aging knowledge scores, attitudes toward older adults, and willingness to pursue a career in geriatric nursing. For all continuous variables, data normality was assessed using the Kolmogorov–Smirnov test, supported by skewness, kurtosis, and histogram visual inspection. Following this assessment, inferential analyses were performed with appropriate parametric tests.

One-way ANOVA with post-hoc Tukey tests examined group differences in career intentions in geriatric nursing. Categorical variables were compared using chi-square tests, while continuous variables were evaluated with t-tests. Variables demonstrating statistical significance in univariate analysis were included as independent variables. The dependent variable was nursing students’ willingness to pursue a career in geriatric nursing. A binary logistic regression (backward method) was then performed, given the presence of dummy variables. Statistical significance was set at *P* < 0.05, with a 95% confidence level.

### Ethical consideration

Ethical approval for the study was granted by the Hunan Traditional Chinese Medical College Ethics Committee (No. YXLL2024-06005). Institutional permissions were secured from the five selected schools. Participants received an information sheet detailing the study’s purpose, procedures, and duration. Informed consent was obtained from students who agreed to participate. Additionally, participation was voluntary, and students could withdraw at any time before survey submission. Furthermore, responses were anonymous, and confidentiality of personal information was maintained by the researchers.

## Result

### Demographic characteristics

Of the 3,762 responses obtained, 140 (3.72%) were excluded primarily due to incomplete data or completion in under five minutes. This resulted in 3622 valid questionnaires, yielding a response rate of 96.27%. Vocational nursing students were aged 17–24 (Mean = 19.11, SD = 1.05). Most participants were female (83.69%, *n* = 3031), second-year students (49.17%, *n* = 1781), and r*u*ral residents (80.73%, *n* = 2924). The vocational nursing students originated from five provinces in Central-South China. The distribution of participants by province of school was as follows: Hunan (*n* = 772, 21.31%), Henan (*n* = 747, 20.62%), Hubei (*n* = 716, 19.77%), Guangdong (*n* = 695, 19.19%), and Guangxi (*n* = 692, 19.11%).

Additionally, 14.80% (*n* = 536) were only children, and 30.45% (*n* = 1103) did not choose nursing as their first career. Approximately 90% (*n* = 3254) of students reported having good relationships with older adults. Nearly 80% (*n* = 2893) had lived with older adults for at least six months, with 52.13% (*n* = 1888) primarily received care from grandparents during childhood. 67.61% (*n* = 2449) had volunteered experience with the elderly, and 22.78% (*n* = 825) had nursing home experience. Despite 80.78% (*n* = 2926) supporting geriatric nursing courses, only 48.76% (*n* = 1766) had received geriatric nursing training. Demographic data are summarized in Table 1.

**Table 1:** Demographic characteristics of the vocational nursing students (*n* = 3622)

Variables	*n*(%)	Variables	*n*(%)
**Gender**		**Relationship with the elderly**	
Male	591(16.31)	Bad	368(10.16)
Female	3031(83.69)	Good	3254(89.84)
**Year of study**		**Experience of living with the elderly**	
First-year	871(24.05)	No	729(20.13)
Second-year	1781(49.17)	Yes	2893(79.87)
Third-year	970(26.78)	**Grandparental care in childhood**	
**Province of school**		No	1734(47.87)
Henan	747(20.62)	Yes	1888(52.13)
Hubei	716(19.77)	**Volunteering with the elderly**	
Hunan	772(21.31)	No	1173(32.39)
Guangdong	695(19.19)	Yes	2449(67.61)
Guangxi	692(19.11)	**Experiencing working in a nursing home**	
**Residential area**		No	2797(77.22)
Rural	2924(80.73)	Yes	825(22.78)
Urban	698(19.27)	**Perceived need for geriatric nursing courses**	
**Only child at home**		No	696(19.22)
No	3086(85.20)	Yes	2926(80.78)
Yes	536(14.80)	**Actual receipt of geriatric care education**	
**Nursing as a first career choice**		No	1856(51.24)
No	1103(30.45)	Yes	1766(48.76)
Yes	2519(69.55)		

### Career intentions in geriatric nursing, aging knowledge, and attitudes toward older people

Of the 3622 vocational nursing students surveyed, 48.67% (*n* = 1763) expressed career intentions in geriatric nursing after graduation. Scores on the Facts on Aging Quiz (FAQ1), assessing knowledge of aging, ranged from 0 to 22, with a mean of 10.82 (SD = 3.38), no student achieved a perfect score. KAOP scores, on the other hand, varied from 80 to 220, averaging 163.08 (SD = 19.52). This mean score exceeds the theoretical median of 136, indicating a tendency to view the elderly positively.

### Univariate analysis of vocational nursing students’ willingness to pursue a career in geriatric nursing

Our analysis showed no statistically significant differences in nursing students’ willingness to practice geriatric care based on gender, province of school, residential area, and only child at home. However, geriatric nursing intentions were associated with multiple factors. Lower year of study (χ^2^ = 18.500, *P* < 0.001) and nursing as a first career choice (χ^2^ = 17.483, *P* < 0.001) predicted greater willingness. Experience of living with the elderly (χ^2^ = 82.938, *P* < 0.001), positive relationships with older adults (χ^2^ = 79.678, *P* < 0.001), caregiving by grandparents in childhood (χ^2^ = 81.935, *P* < 0.001), volunteering experience with the elderly (χ^2^ = 64.393, *P* < 0.001), and nursing home experience (χ^2^ = 49.136, *P* < 0.001) correlated positively with willingness. Geriatric care training (χ^2^ = 79.904, *P* < 0.001) and perceived need for specialized courses (χ^2^ = 98.752, *P* < 0.001) were linked to increased willingness. Stronger attitudes (*t* = -7.395, *P* < 0.001) and higher knowledge (*t* = -20.150, *P* < 0.001) distinguished intending students from non-intending peers (Table 2).

**Table 2:** Comparison of career intentions in geriatric nursing by sociodemographic and experiential factors (n = 3622)

Variables	Non-willingness	Willingness	χ^2^/t	*P*	
**Gender, n(%)**			0.176	0.354	
Male	308(52.12)	283(47.88)			
Female	1551(51.17)	1480(48.88)			
**Year of study, n(%)**			18.5	< 0.001	
First-year	432(49.60)	439(50.40)			
Second-year	872(48.96)	909(51.04)			
Third-year	555(57.22)	415(42.78)			
**Province of school, n(%)**			4.301	0.367	
Henan	393(52.61)	354(47.39)			
Hubei	354(49.44)	362(50.56)			
Hunan	416(53.89)	356(46.11)			
Guangdong	347(49.93)	348(50.07)			
Guangxi	349(50.43)	343(49.57)			
**Residential area, n(%)**			0.004	0.492	
Rural	1500(51.30)	1424(48.70)			
Urban	359(51.43)	339(48.57)			
**Only child at home, n(%)**			0.726	0.21	
No	1593(51.62)	1493(48.38)			
Yes	266(49.63)	270(50.37)			
**Nursing as a first career choice, n(%)**			17.483	< 0.001	
No	624(56.57)	479(43.43)			
Yes	1235(49.03)	1284(50.97)			
**Relationship with the elderly, n(%)**			79.678	< 0.001	
Bad	270(73.37)	98(26.63)			
Good	1589(48.83)	1665(51.17)			
**Experience of living with the elderly, n(%)**			82.938	< 0.001	
No	484(66.40)	245(33.60)			
Yes	1375(47.53)	1518(52.47)			
**Grandparental care in childhood, n(%)**			81.935	< 0.001	
No	1026(59.17)	708(40.83)			
Yes	833(44.12)	1055(55.88)			
**Volunteering with the elderly, n(%)**			64.393	< 0.001	
No	715(60.95)	458(39.05)			
Yes	1144(46.71)	1305(53.29)			
**Experiencing working in a nursing home, n(%)**			49.136	< 0.001	
No	1524(54.49)	1273(45.51)			
Yes	335(40.60)	490(59.40)			
**Perceived need for geriatric nursing courses, n(%)**			98.752	< 0.001	
No	475(68.25)	221(31.75)			
Yes	1384(47.30)	1542(52.70)			
**Actual receipt of geriatric care education, n(%)**			79.904	< 0.001	
No	1087(58.57)	769(41.43)			
Yes	772(43.71)	994(56.29)			
**FAQ 1, Mean(SD)**	9.78(3.34)	11.92(3.05)	-20.150*	< 0.001	
**Total KAOP, Mean(SD)**	160.76(18.79)	165.53(19.98)	-7.395*	< 0.001	

Note: * denotes the t-value, and other values are Chi-square (χ^2^) values

Abbreviations: FAQ1, Facts on AgingQuiz1; KAOP, Kogan’s Attitudes towards Older People Scale

### Multivariate analysis of factors influencing vocational nursing students’ willingness to pursue a career in geriatric nursing

Binary logistic regression was employed to identify factors associated with vocational nursing students’ willingness to pursue a career in geriatric nursing. The dependent variable was the students’ willingness to pursue a career in geriatric nursing, with significant variables from univariate analysis included as independent variables. Binary logistic regression identified associated factors of geriatric nursing intentions (*P* < 0.05, Table 3, Nagelkerke R² = 0.213): sociodemographic factors (lower year of study, nursing as a first career choice), experiential factors (experience of living with older adults, relationship with the elderly, childhood caregiving, geriatric training, need for geriatric courses, volunteering, nursing home experience), and stronger attitudes and knowledge. Odds ratios and confidence intervals are detailed in Table 3.

**Table 3:** Logistic regression analysis of factors influencing geriatric nursing career intentions among vocational students (*n* = 3622)

Variables	B	SE	OR	95% CI		*P*
				Lower	Upper	
Year of study	-0.308	0.053	0.735	0.663	0.815	< 0.001
Nursing as a first career choice	0.259	0.082	1.296	1.104	1.520	0.002
Relationship with the elderly	0.443	0.138	1.557	1.187	2.042	0.001
Experience of living with the elderly	0.252	0.109	1.286	1.039	1.593	0.021
Grandparental care in childhood	0.305	0.083	1.356	1.152	1.597	< 0.001
Volunteering with older adults	0.397	0.081	1.488	1.270	1.742	< 0.001
Experiencing working in a nursing home	0.397	0.091	1.487	1.245	1.775	< 0.001
Perceived need for geriatric nursing courses	0.292	0.104	1.339	1.093	1.641	0.005
Actual receipt of geriatric care education	0.184	0.079	1.202	1.030	1.403	0.019
FAQ 1	0.186	0.013	1.205	1.175	1.235	< 0.001
Total KAOP	0.012	0.002	1.012	1.008	1.015	< 0.001

## Discussion

The primary findings of this study offer novel insights into the career intentions of vocational nursing students in Central-South China. Among the 3622 participants, a substantial proportion (48.67%) expressed an intention to pursue geriatric nursing, despite the increasing demand for professionals in this field. This rate exceeds the 38.1% reported among Chinese undergraduate nursing students [19] and the willingness rate among 2,250 undergraduate nursing students across nine countries [27]. This relatively high figure is particularly noteworthy, underscoring the value of this study in examining the vocational cohort, who are primarily oriented toward essential grassroots healthcare roles in China’s rapidly aging society. Despite this positive indication and the students’ generally positive attitudes toward older adults, this intention rate remains below the global average of approximately 50% [28] and is insufficient to meet the local workforce demand. The fact that over half (51.33%) of the students remain unwilling highlights a persistent and complex challenge, suggesting that while initial interest exists, systemic or perceived barriers continue to deter a critical segment of this potential workforce from specializing in geriatric care.

Our study found that year of study and nursing as a first career choice were key factors influencing vocational nursing students’ intent to enter geriatric care. Third-year students exhibited lower intentions, possibly due to exposure to diverse specialties [29]. This may be attributed to broader clinical exposure among senior students. They often develop interests in specialties like acute care, perceived as more dynamic [29]. Conversely, choosing nursing as a first career when applying for college reflects a commitment to the profession, which may align with societal expectations of caregiving roles, thereby enhancing intentions [30]. These findings suggest that early career orientation is pivotal for vocational nursing students’ preferences. This emphasis on commitment aligns with recent international findings that highlight the importance of established life commitment and maturity in shaping career choice, suggesting a robust connection between commitment and intention in the geriatric field [31].

In contrast to previous studies suggesting that female students are more inclined toward geriatric nursing [32, 33], this study found no significant gender differences in vocational nursing students’ geriatric nursing intentions. This inconsistency may reflect China’s vocational training culture or sample composition and warrants further investigation [34]. This warrants further exploration to understand contextual influences.

Educational and knowledge-based factors are crucial determinants explaining the low geriatric career intention rate among vocational nursing students. Our study reported a low knowledge level, consistent with findings that nursing students lack adequate knowledge about aging [35, 36]. This observed knowledge deficit exists despite geriatric nursing being a mandatory theoretical component of the core Chinese nursing curriculum [37]. This contradiction points to significant structural deficiencies specific to the vocational track, which are often rooted in the course’s status as an elective in some vocational institutions or its insufficient allocation of dedicated clinical hours [38]. This educational gap, further indicated by the need for geriatric nursing courses as a significant predictor, likely reduces students’ confidence in managing older adults’ complex needs and consequently lowers their career intentions [39]. Furthermore, the academic timing of the specialized module (typically offered in the second year) may contribute to the complex relationship between academic progress and career intent: while sophomore students showed a slightly higher intention than freshmen, the intention among juniors (final year) dropped significantly. This pattern suggests that limited early exposure is often negated by negative perceptions gained from broader clinical placements in later years. Based on our findings, vocational nursing schools should consider systematically integrating geriatric nursing modules into the core curriculum rather than offering them as electives. Crucially, the timing and mandatory status of course implementation are pivotal; these modules should be introduced early in the curriculum and maintained as mandatory to combat the negative influence of subsequent, broader clinical exposure. Simulation-based training and structured geriatric clinical placements could enhance students’ perceived preparedness and confidence in geriatric care [40].

Our study reveals that the attitudes of vocational nursing students toward older adults significantly influence their intent to pursue geriatric care. Students in Central-South China exhibited more positive attitudes (KAOP mean = 163.08) than those in the Northeast (KAOP mean = 140.41) [22]. This regional disparity likely stems from cultural differences in family structures and community interactions [19]. Frequent intergenerational contact, which may be more prevalent in Central-South China, fosters respect for the elderly and enhances students’ willingness to engage in geriatric care [41]. Unlike formal professional contact, the prevalence of intergenerational contact in daily life—such as living with older adults or receiving grandparental care—serves as a cultural mechanism that fosters emotional bonds and reinforces values of filial piety [42]. These early, informal interactions mitigate age-based bias and enhance psychological readiness to engage with aging populations [43]. These findings align with studies from Taiwan and Hong Kong, suggesting that such collectivist values play a pivotal role in shaping positive perceptions [44, 45]. However, ageism remains a persistent barrier that can counteract these cultural advantages [46]. Consequently, educational strategies must go beyond skills training to explicitly address ageist stereotypes, leveraging these cultural strengths to nurture a genuine willingness to care.

Consistent with prior research [29, 46], our results reveal that specific professional experiences—namely volunteering and nursing home internships—significantly shape students’ career intentions. Distinct from familial contact, these structured vocational experiences increase empathy and technical familiarity, effectively demystifying the professional role of a geriatric nurse [47, 48]. Our analysis suggests that clinical exposure exerts a dual influence. While positive professional engagement nurtures motivation, unguided confrontation with systemic realities—such as heavy workloads, low pay, and emotional burnout—can deter prospective nurses rather than recruit them [42, 49]. Therefore, mere exposure is insufficient. Vocational programs should implement pedagogical scaffolding, combining structured service-learning with mentorship from experienced practitioners. This supportive framework is essential for helping students navigate these professional realities, ensuring that their initial interest translates into sustained career commitment.

These diverse influences are integrated through TPB. Positive KAOP scores (OR = 1.012, 95% CI: 1.008–1.015, *P* < 0.001) reflect favorable attitudes toward geriatric nursing, shaping stronger intentions. Subjective norms, shaped by cultural values such as filial piety, integrate familial and professional influences, such as caregiving experiences or commitment to nursing [42, 50]. Perceived behavioral control is enhanced by exposure to geriatric courses, clinical placements, and volunteering (OR = 1.488, 95% CI: 1.270–1.742, *P* < 0.001), which bolster confidence and competence. Thus, TPB elucidates how attitudinal, cultural, and experiential factors converge to influence geriatric nursing intentions.

Our study highlights the multifaceted factors influencing vocational nursing students’ intentions to work in geriatric care. Positive attitudes, meaningful clinical experiences, and cultural values enhance intentions, while knowledge gaps and a higher year of study pose challenges. Future research should adopt longitudinal designs to establish causality. Additionally, qualitative methods could uncover reasons for the suboptimal intention rate [51]. Exploring organizational factors, such as workplace support, may further enrich the understanding of students’ career intentions [39]. Cross-cultural comparisons between vocational and undergraduate nursing students could also help elucidate the role of contextual influences [52, 53].

## Strengths and limitations

This study, among the first to examine vocational nursing students’ intent to enter geriatric nursing in Central-South China, provides significant insights. It used a large, diverse sample (*n* = 3622) across five provinces, ensuring robust representation. In addition, the 96.3% response rate and culturally adapted FAQ1 and KAOP scales, with adequate reliability, strengthen the findings’ credibility. Furthermore, multivariable logistic regression adjusted for confounding factors. This approach enhanced the validity of our results. The Theory of Planned Behavior serves as a robust framework for this research.

However, several limitations warrant consideration. The cross-sectional design prevents causal inferences, limiting our ability to determine temporal relationships. Furthermore, the relatively low explanatory power of the final regression model (Nagelkerke R² = 0.213) suggests that the measured variables only account for a small proportion of the variance in career intention. This limitation is typical of complex psychological outcomes, and it highlights the influence of unmeasured external factors, such as specific regional employment policies, long-term salary expectations, perceived social status, or institutional support, which are known to influence vocational choice but were not included in the current model. Additionally, self-reported measures are susceptible to biases, such as recall inaccuracies or social desirability effects. China’s unique cultural and educational context may limit generalizability to other regions. Therefore, future longitudinal studies and culturally refined scales could further enhance understanding of these intentions.

## Conclusion

This study fills a critical gap by examining vocational nursing students’ intentions to pursue geriatric care in Central-South China. Few studies have addressed this workforce challenge in the region, making these findings timely and relevant. The research reveals a moderate intention rate (48.67%) among 3622 students. Eleven factors across sociodemographic, experiential, and attitudinal-knowledge domains shape career intentions, accounting for 21.3% of variation (Nagelkerke R² = 0.213).

Thus, the Theory of Planned Behavior provides a robust framework for elucidating how attitudes, social influences, and perceived behavioral control shape students’ career decisions. Multivariable logistic regression further strengthens the analysis by identifying key predictors, enhancing the study’s methodological rigor. Despite positive attitudes (KAOP mean = 163.08), low knowledge levels (FAQ1 mean = 10.82) limit students’ understanding of geriatric care needs, hindering stronger intentions. Therefore, curricular reform is essential to address these knowledge gaps. For example, integrating gerontological modules, expanding clinical placements, and fostering intergenerational contact can target attitudes and nurture motivation, preparing students for geriatric care.

Looking forward, our findings guide the design of targeted interventions to address workforce shortages in aging societies. Future longitudinal studies should explore how sustained educational reforms impact career trajectories and evaluate their effectiveness.

## Supplementary Information

Below is the link to the electronic supplementary material.

Supplementary Material 1

## Data Availability

Databased: The de-identified dataset generated and analyzed during the current study is available in the Open Science Framework (OSF) repository, DOI: 10.17605/OSF.IO/6XCDM.

## Abbreviations

TPB: Theory of Planned Behavior
EPV: Events Per Variable
FAQ1: Facts on Aging Quiz 1
CVI: content validity index
KAOP: Kogan’s Attitudes towards Older People
SD: Standard Deviation
