# Supplementary material for: Factors influencing vocational nursing students’ career intentions in geriatric nursing: a cross-sectional study
Source: BMC Nurs. 2026 Jan 28;25:167. doi: 10.1186/s12912-026-04346-z (PMC12924232; doi:10.1186/s12912-026-04346-z)
Supplement: Supplementary file 1 — Supplementary Material 1 [file 12912_2026_4346_MOESM1_ESM.pdf]

Additional file 1: Demographic Questionnaire

Project Title: Factors Influencing Vocational Nursing Students' Career Intentions in Geriatric Nursing: A Cross-Sectional Study

Instruction: This questionnaire is designed for academic research purposes. Your participation is entirely voluntary and all data will remain strictly anonymous and confidential. Please select the most appropriate option for each question based on your actual situation.

Part I: Sociodemographic Characteristics

1. **Age:** _______ years
2. **Gender:**
   - ○ Male
   - ○ Female
3. **Year of study:**
   - ○ First-year
   - ○ Second-year
   - ○ Third-year
4. **Residential Area:**
   - ○ Urban
   - ○ Rural
5. **In which province is your school located?**
   - ○ Hubei
   - ○ Hunan
   - ○ Guangdong
   - ○ Guangxi
   - ○ Henan
6. **Are you an only child at home?**
   - ○ Yes
   - ○ No
7. **Was nursing your first career choice when applying for college?**
   - ○ Yes
   - ○ No

Part II: Experiential Factors

8. **How would you describe your general relationship with the elderly?**
   - ○ Bad
   - ○ Good
9. **Have you ever lived with the elderly (e.g., grandparents or other elderly relatives) for at least six months?**
   - ○ Yes
   - ○ No
10. **Did you receive care from your grandparents during your childhood?**
    - ○ Yes
    - ○ No
11. **Have you ever had volunteer experience with the elderly?**
    - ○ Yes
    - ○ No
12. **Did you have any prior work experience in a nursing home (e.g., internships or part-time jobs)?**
    - ○ Yes
    - ○ No

Part III: Educational Factors

13. **Do you perceive a need for specialized geriatric nursing courses?**
    - ○ Yes
    - ○ No
14. **Have you received any geriatric nursing education or training?**
    - ○ Yes
    - ○ No

Part IV: Career Intention

15. **Would you consider working with older adults upon graduation?**
    - ○ Willingness
    - ○ Non-willingness
